# The effect of calorie restriction on insulin signaling in skeletal muscle and adipose tissue of Ames dwarf mice

**DOI:** 10.18632/aging.100700

**Published:** 2014-10-05

**Authors:** Denise S. Wiesenborn, Vinal Menon, Xu Zhi, Andrew Do, Adam Gesing, Zhihui Wang, Andrzej Bartke, Deborah A. Altomare, Michal M. Masternak

**Affiliations:** ^1^ Burnett School of Biomedical Sciences, College of Medicine, University of Central Florida, Orlando, FL 32827, USA; ^2^ Department of Cell Biology and Anatomy, School of Medicine, University of South Carolina Columbia, SC 29209, USA; ^3^ Center for Reproductive Medicine, Department of Obstetrics and Gynecology, Peking University Third Hospital, Beijing 100191, China; ^4^ Department of Oncological Endocrinology, Medical University of Lodz, 90-752 Lodz, Poland; ^5^ Department of Internal Medicine, Southern Illinois University School of Medicine, Springfield, IL 62794, USA; ^6^ Department of Head and Neck Surgery, The Greater Poland Cancer Centre, 61-866 Poznan

**Keywords:** Ames dwarf, insulin, adipose tissue, skeletal muscle, adiponectin, obesity

## Abstract

Long-living Ames dwarf (df/df) mice are homozygous for a mutation of the *Prop1^df^* gene. As a result, mice are deficient in growth hormone (GH), prolactin (PRL) and thyrotropin (TSH). In spite of the hormonal deficiencies, df/df mice live significantly longer and healthier lives compared to their wild type siblings. We studied the effects of calorie restriction (CR) on the expression of insulin signaling genes in skeletal muscle and adipose tissue of normal and df/df mice. The analysis of genes expression showed that CR differentially affects the insulin signaling pathway in these insulin target organs. Moreover, results obtained in both normal and Ames dwarf mice indicate more direct effects of CR on insulin signaling genes in adipose tissue than in skeletal muscle. Interestingly, CR reduced the protein levels of adiponectin in the epididymal adipose tissue of normal and Ames dwarf mice, while elevating adiponectin levels in skeletal muscle and plasma of normal mice only.

In conclusion, our findings suggest that both skeletal muscle and adipose tissue are important mediators of insulin effects on longevity. Additionally, the results revealed divergent effects of CR on expression of genes in the insulin signaling pathway of normal and Ames dwarf mice.

## INTRODUCTION

Ames dwarf (df/df) mice are homozygous for a spontaneous recessive mutation of the prophet of pituitary factor-1 (*Prop1*) gene, which inhibits development of three specific anterior pituitary cell types - somatotrophs, lactotrophs and thyrothrophs [1]. The absence of these cell types in the df/df mice leads to deficiency of growth hormone (GH), prolactin (PRL) and thyrotropin (TSH). Interestingly, however, these mutants live significantly longer (40-60%) and healthier lives compared to their normal siblings. Ames dwarf mice phenotypically appear normal at birth but they grow at a slower rate and reach only half of the normal adult body weight, compared to their normal littermates. In addition, their sexual maturity is delayed and females are sterile because of PRL deficiency [2, 3]. These mutant mice also exhibit very low levels of insulin-like growth factor-1 (IGF-1) and thyroid hormones [2]. Furthermore, they have a reduced body temperature [4], but their food and oxygen consumption per gram of body weight are increased [5, 6]. Ames dwarf mice are less prone to cancer [7]. These mutants show increased insulin sensitivity and glucose tolerance [8], thus displaying no diabetic phenotype. Furthermore, Ames dwarf mutants have reduced response of skeletal muscle to high levels of insulin, which might be important for their control of glucose homeostasis and as well their positive effects in extended longevity [9].

Calorie restriction (CR), is the only efficient intervention which delays aging and extends lifespan [10]. Laboratory animals subjected to reduced caloric intake, exhibit a number of beneficial effects including extension of lifespan, reduced body weight, plasma glucose and insulin levels; and improved insulin sensitivity and health span [11]. Studies in various animal species revealed that CR delays aging, decreases cholesterol levels and blood pressure [12]. Studies involving mice and rats support the concept that CR delays the aging process and reduces the incidence of several age-related diseases including type 2 diabetes and cancer [13]. In addition, it has been shown that the prolongation of life can be greater than 40% in mice under CR regimen; with even greater extension of longevity in non-mammalian models [14, 15]. Some investigations, at least in rats, suggest that CR delays aging by retarding and rescuing the age-related increase of mortality [16]. It was shown that a CR diet not only extends lifespan in rodents, but also in other model organisms including rhesus monkeys, *Drosophila melanogaster, Caenorhabditis elegans* and yeast [17]. Moreover, studies with CR regimen in humans indicate various health benefits, including decreased body weight and adiposity [18], protection against atherosclerosis [12], enhancement of memory in older people [19], as well as improvement of cardiac function [20]. Existing data indicate that the effect of CR on the aging process might be mediated by a reduction in glycosylation and hormetic responses [21], oxidative damage, metabolic adjustments or changes in specific gene expression [22]. Interestingly, Ames dwarf mice with increased lifespan and better insulin sensitivity and glucose tolerance show similar characteristics to normal mice subjected to calorie restriction, but these mutants are not CR mimetics [5]. Furthermore, these long living dwarf mice exhibit a further extension of longevity when subjected to CR [23].

Adipose tissue, is addition to being a fat depot is also an active endocrine organ, producing and secreting different peptide hormones (adipokines) including adiponectin [24]. Adiponectin is an adipokine hormone and is secreted mainly by adipose tissue [25]. This plasma hormone regulates insulin sensitivity, glucose usage and energy homeostastis [26]. Moreover, adiponectin plays a key role in the suppression of metabolic disorders and a reduction in adiponectin levels appears to contribute to the consequences of type 2 diabetes [27], obesity and atherosclerosis [28]. The levels of adiponectin are lower in obese than in lean subjects and are reduced in association with insulin resistance and type 2 diabetes. Present research in the field of obesity and diabetes highlights the fact that pro-inflammatory cytokines are known to downregulate adiponectin levels [29].

Based on extensive studies of CR and *Prop1* mutation on insulin signaling, metabolism and aging there is some evidence that indicates that Ames dwarfism and CR may act through similar mechanisms but they are certainly not identical [2, 5]. It was also shown that CR differently regulates insulin signaling genes in the liver of N and df/df mice [30], the main organ regulating glucose metabolism. Our previous studies indicated significant responses of liver to CR as well as different regulatory mechanisms of insulin signaling pathway between GH-deficient df/df, GH-resistant GHRKO and N mice indicating important role of GH signal in insulin action in the liver.[30, 31]. However, it is known that CR provide systemic not only local benefits improving whole body insulin sensitivity indicating that other important insulin signaling organs may be also affected. Based on this knowledge the purpose of this research was to characterize the interactive effects of CR and the df/df genotype on the insulin signaling pathway in skeletal muscle and epididymal adipose tissue as main insulin target organs and to determine the alterations in the levels of adiponectin in plasma, skeletal muscle and adipose tissue in response to CR.

## RESULTS

At the age of 3 months, df/df and N male mice were randomly divided into four experimental groups, with 10 animals per group: normal mice with unlimited food access (N-AL), normal mice subjected to 30% CR (N-CR), Ames dwarf mice with unlimited food access (df/df-AL), Ames dwarf mice subjected to 30% CR (df/df-CR). Calorie restriction was continued for approximately 8 months and fasted animals were sacrificed at the age of 12 months and tissues were collected.

### The effect of CR on body weight and fasting blood glucose

**Figure 1:**
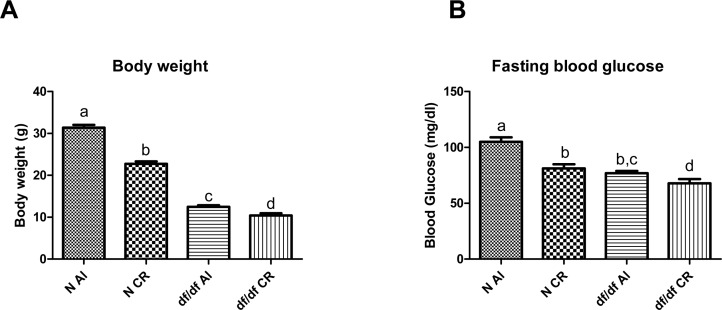
The effect of CR on bodyweight (**A**) and fasting blood glucose (**B**) of Normal (N) and Ames dwarf (df/df) mice fed ad libitum (AL) or subjected to 30% calorie restriction (CR). Groups which do not share the same letter display a statistical significance (p < 0.05).

As expected, nine months of 30% calorie restriction caused a decrease in body weight in N-CR and df/df-CR mice compared to genotype matched controls fed *ad libitum* (AL) (P=0.0001 and P=0.0008, respectively) (Figure 1A).

Fasting blood glucose analysis indicated significant decrease of circulating glucose levels in df/df-AL mice compared to N-AL mice (P=0.0001). Calorie restriction caused a significant decrease in blood glucose levels in N-CR mice relative to N-AL littermates (P=0.0001) and it also led to decreased blood glucose levels in df/df-CR mice compared to df/df-AL (P=0.0151) (Figure 1B).

### The effect of CR on the expression of insulin signaling genes in skeletal muscle

**Figure 2:**
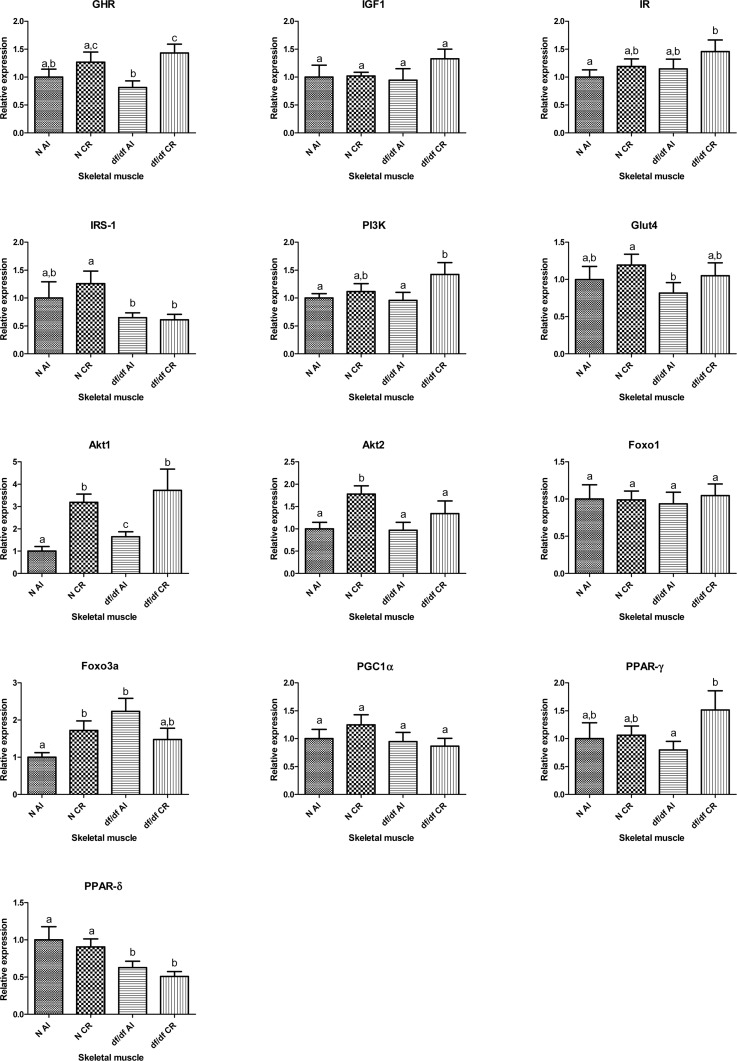
Relative gene expression in skeletal muscle of Normal (N) and Ames dwarf (df/df) mice fed ad libitum (AL) or subjected to 30% calorie restriction (CR). Groups which do not share the same letter display a statistical significance (p < 0.05).

The mRNA expression levels of growth hormone receptor (GHR) and insulin like growth factor 1 (IGF-1) were not affected by the genotype in skeletal muscle of df/df mice. However, expression of GHR at the mRNA level was significantly affected by the diet; without significant interaction (P=0.0064). Calorie restriction increased the mRNA expression of GHR in df/df-CR mice when comparing to df/df-AL (P=0.0028) (Figure 2). Two way ANOVA analysis did not show any significant effects of either CR or genotype on transcript levels of insulin receptor (IR), phosphoinositide 3-kinase (PI3K) or glucose transporter 4 (GLUT4). However, t-test analysis indicated increased level of PI3K mRNA in df/df-CR relative to df/df-AL littermates (P=0.0418). The expression of insulin receptor substrate 1 (IRS1) mRNA was significantly affected by genotype (P=0.0141), without evidence of significant interaction between diet and genotype. The expression levels of both PKB-alpha serine/threonine-protein kinase (Akt1) and PKB-beta serine/threonine-protein kinase (Akt2) were significantly changed by the diet (P=0.0002 and P=0.0082, respectively) (Figure 1). Transcript levels of Akt1 were elevated in df/df-AL relative to N-AL controls (P=0.0261). CR increased the mRNA levels of Akt 1 in N-CR and df/df CR animals compared to matching AL controls (P=0.0001 and P=0.0205, respectively). The expression of Akt2 was significantly increased by CR only in N mice (P=0.0023) (Figure 2).

The expression of forkhead box O1 (FOXO1) and forkhead box O3A (FOXO3a) was not affected by either genotype or CR. Two way ANOVA indicated significant diet and genotype interaction for FOXO3a (P=0.0111). Calorie restriction increased expression of FOXO3a in N-CR mice compared to N-AL animals (P=0.0134). The level of FOXO3a was upregulated in df/df-AL mice relative to N-AL mice (P=0.0025), and CR normalized the expression of this transcription factor in df/df mice (Figure 2).

There was no evidence for significant effect of diet, genotype or interaction at the level of peroxisome proliferator-activated receptor gamma coactivator 1-alpha (PGC1α) and peroxisome proliferator-activated receptor gamma (PPARγ) mRNA expression. The mRNA expression of PPARγ was increased by CR only in df/df mice (P=0.0334). However, peroxisome proliferator-activated receptor delta (PPARδ) mRNA was significantly affected by the genotype (P=0.002) with suppressed expression levels in skeletal muscle of df/df mice (Figure 2).

### The effect of CR on insulin signaling genes in epididymal adipose tissue

The expression levels of GHR, IGF-1, IR, IRS1 GLUT4, Akt1, Akt2, FOXO1, FOXO3a, PGC1α, PPARγ and PPARδ mRNAs were affected by the diet (P=0.0001, P=0.016, P=0.0001, P=0.0002, P=0.0001, P=0.005, P=0.0001, P=0.0014, P=0.0281, P=0.0094, P=0.0001 and P=0.0003, respectively). At the same time genotype affected the expression level of GHR, IGF-1, IRS-1, GLUT4, Akt2, FOXO3a, PPARγ and PPARδ (P=0.0001, P=0.0001, P=0.0006, P=0.0001, P=0.0001, P=0.0039, P=0.0022 and P=0.0001, respectively).

**Figure 3:**
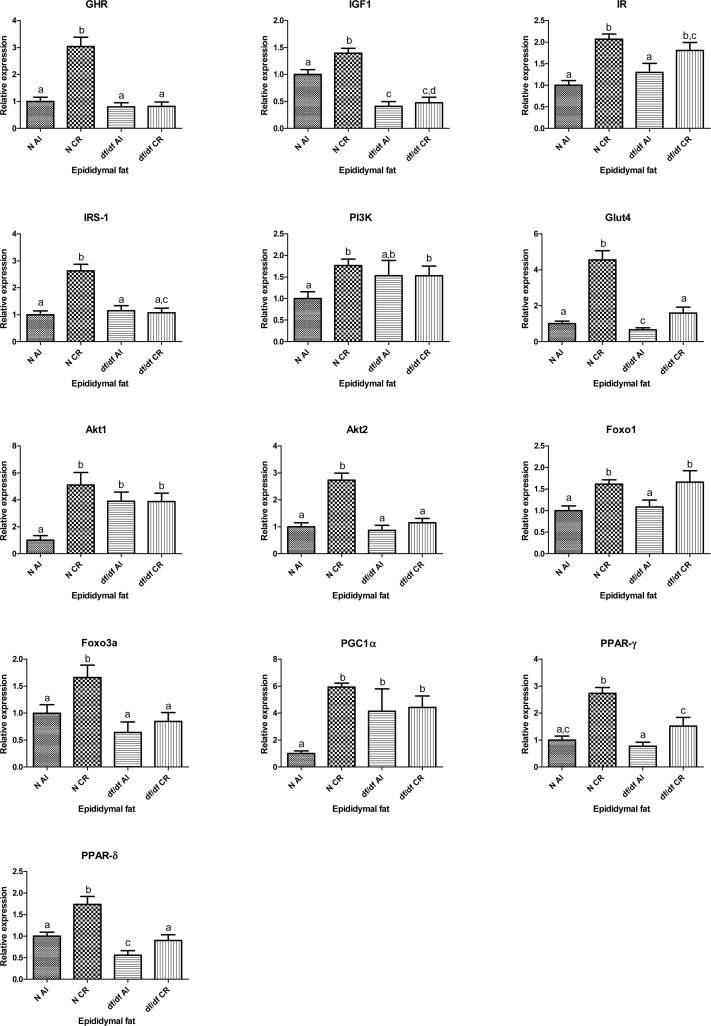
Relative gene expression in epididymal adipose tissue of Normal (N) and Ames dwarf (df/df) mice fed ad libitum (AL) or subjected to 30% calorie restriction (CR). Groups which do not share the same letter display a statistical significance (p < 0.05)

Importantly, there was a significant interaction between diet and genotype for transcript levels of GHR, IRS-1, Akt1, Akt2, GLUT4, PGC1α and PPARγ (P=0.0001, P=0.0001, P=0.0043, P=0.0007, P=0.0002, P=0.0195, P=0.0297, respectively). Additional t-test analyses showed significantly increased mRNA expression levels of GHR, IGF-1, IRS-1, PI3K, Akt1, Akt2, PGC1α and FOXO3a in epididymal adipose tissue in N-CR mice compared to their N-AL controls (P=0.0001, P=0.0029, P=0.0001, P=0.0014, P=0.0003, P=0.0001, P=0.0001, P=0.0148, respectively) with no alterations in df/df-CR when compared to df/df-AL. Expression levels of IR, GLUT4, FOXO1, PPARγ and PPARδ were significantly increased in epididymal adipose tissue of N-CR compared to N-AL mice (P= 0.0001, P=0.0001, P=0.0003, P= 0.0001, P=0.0012, respectively). There was also a significant increase in mRNA levels of IR, GLUT4, FOXO1, PPARγ and PPARδ in df/df-CR mice compared to df/df-AL controls (P=0.0429, P=0.0001, P=0.0392, P=0.0257, P=0.0320, respectively). Interestingly, expression of GLUT4 was significantly decreased in df/df-AL mice compared to their N-AL controls (P=0.0377) (Fig. 3).

### The effect of CR on adiponectin levels in epididymal adipose tissue, skeletal muscle and plasma

**Figure 4:**
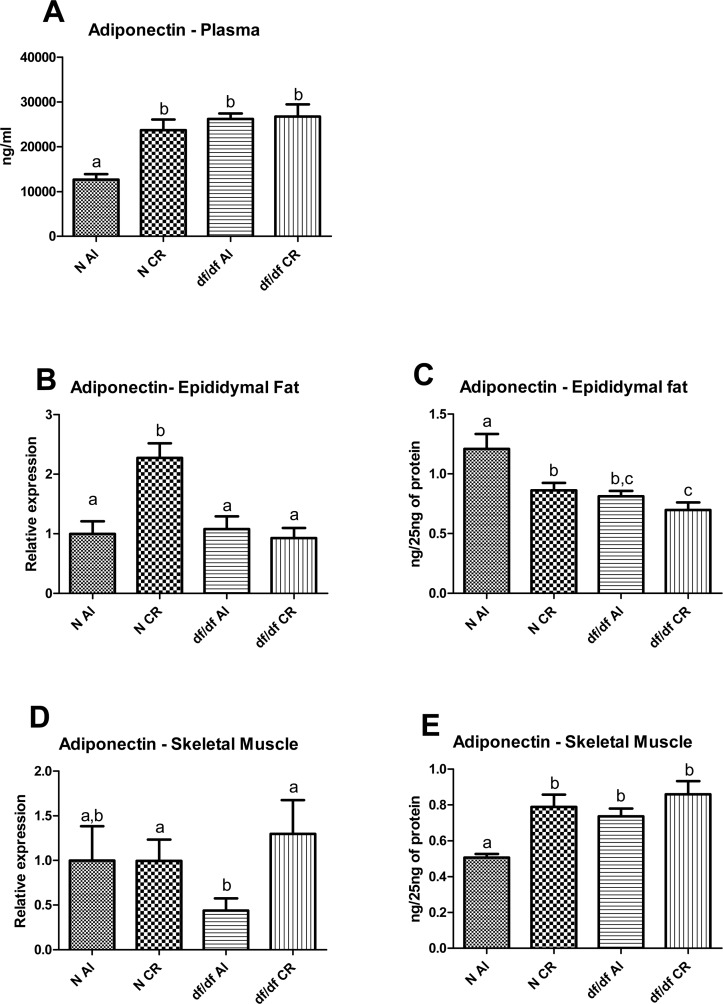
Adiponectin levels in Normal (N) and Ames dwarf (df/df) mice fed ad libitum (AL) or subjected to 30% calorie restriction (CR). (**A**) Plasma adiponcetin, (**B**) mRNA adiponectin in epididymal adipose tissue, (**C**) Protein level of adiponectin in epididymal adipose tissue, (**D**) mRNa adiponectin in skeletal muscle, (**E**) protein level of adiponectin in skeletal muscle. Groups which do not share the same letter display a statistical significance (p < 0.05).

Plasma adiponectin level was significantly affected by diet and genotype (P=0.0038 and P=0.0001, respective-ly), however there was also significant genotype and diet interaction (P=0.0078) (Figure 4A). The df/df-AL mice had higher level of adiponectin protein than N-AL littermates (P=0.0001), while CR increased plasma adiponectin in N mice (P=0.0003) bringing it to the level maintained by df/df mice. However, CR did not alter the level of adiponectin in df/df-CR mice compared to df/df-AL mutants. (Figure 4A).

The mRNA adiponectin level in epididymal fat indicated significant diet and genotype effect (P=0.0048 and P=0.0017, respectively) (Figure 4B). Also, there was significant diet and genotype interaction (P=0.0111). Additional t-test analysis between each experimental group showed significant increase of adiponectin mRNA in epididymal fat from N-CR animals compared to N-AL mice (P=0.0005) with no difference between either df/df-AL and df/df-CR or df/df-AL and N-AL (Figure 4B).

Interestingly the alterations in adiponectin protein levels in adipose tissue did not correspond to the pattern of mRNA expression. Both diet and genotype significantly affected adiponectin level in epididymal fat (P=0.0082 and P=0.0018, respectively) and there was no evidence for diet and genotype interaction (Figure 4C). There was significant downregulation of epididymal fat adiponectin levels in N-CR, df/df-AL and df/df-CR relative to N-AL animals (P=0.0150, P=0.0043 and P=0.0014, respectively) (Figure 4D).

Expression of adiponectin at the mRNA level in skeletal muscle was not affected by either genotype or diet, however, t-test analysis indicated significant difference between df/df-AL and df/df-CR (P=0.0297) (Figure 4D). In contrast, protein level of adiponectin was significantly upregulated by both diet and genotype (P=0.0006 and P=0.0085, respectively) without significant interaction. Further t-test analysis indicated that df/df-AL mice exhibited higher levels of adiponectin protein in skeletal muscle compared to N-AL littermates (P=0.0001). However, CR increased adiponectin levels in N-CR mice relative to N-AL mice (P=0.0003) there is no further increase of this adipokine in df/df-CR mice compared to df/df-AL mice which are already characterized by elevated protein levels of adiponectin in skeletal muscle (Figure 4E).

### The effect of CR and genotype on the size of adipocytes from epididymal adipose tissue

**Figure 5:**
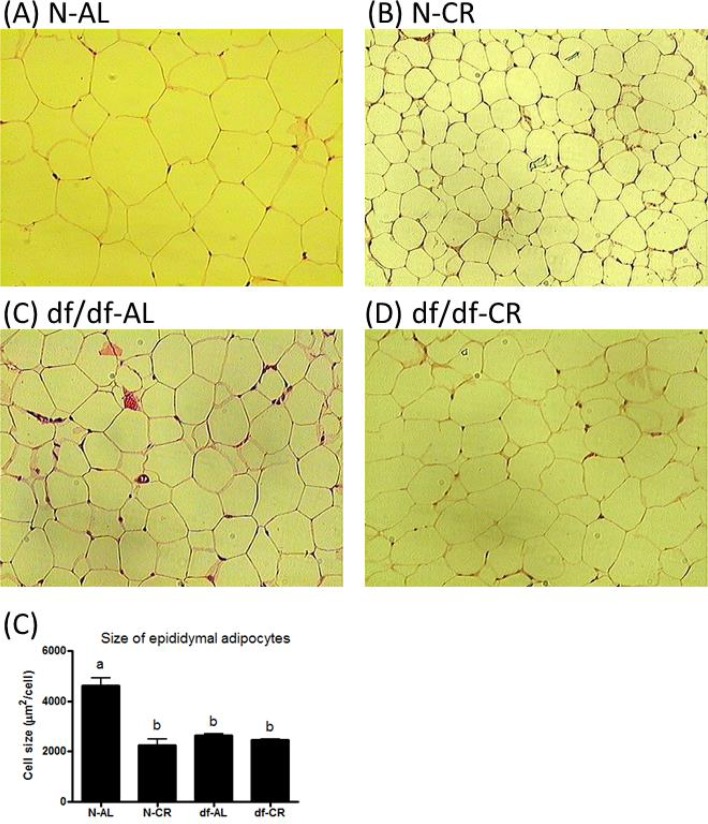
Adipocytes of epididymal adipose tissue from Normal and Ames dwarf mice. **Histological** effect of genotype and CR on adipocytes size of epididymal adipose tissue from ormal and Ames dwarf mice.

The size of adipocytes is strongly related to the release of insulin-sensitizing hormones and whole body insulin sensitivity [32]. Analysis of histological sections revealed a significant, approximately 44%, reduction in the size of adipocytes from df/df-AL mice compared to N-AL littermates (P=0.0005). Calorie restriction significantly decreased the size of adipocytes in N-CR(P=0.0007) but not in df/df-CR mice relative to the corresponding AL controls (Figure 5 A-C).

## DISCUSSION

Ames dwarf mice are well established research models in the field of longevity and aging owing to their extended life expectancy. [2, 23, 33]. Importantly these animals not only live longer but are also healthier, compared to their normal siblings, showing some protection from age-related diseases including cancer and diabetes[7]. These long-living mutant mice are also characterized by high insulin sensitivity and enhanced glucose tolerance. Subjecting Ames dwarf mice to hyperinsulinemic-euglycemic clamp studies showed that there is a multi-organ enhancement of insulin actions and glucose metabolism, with increased glucose uptake by skeletal muscle and visceral adipose tissue in df/df mice compared with N littermates [34].

In this study we investigated the interactive effects of CR and Ames dwarfism on insulin signaling pathway in skeletal muscle and epididymal adipose tissue to determine the mechanism controlling enhanced glucose metabolism in these insulin target organs.

As expected from previous observations, chronic 30% CR in df/df and N mice caused a significant decrease in the body weight; which indicates that food intake was indeed reduced during the experiment [30]. Calorie restriction also decreased fasting blood glucose levels in N animals bringing it to that maintained by healthy df/df mice. Moreover, the diet caused further decrease of already low levels of fasting glucose in df/df mice. This finding corresponds with previously published studies showing an improvement in insulin sensitivity as revealed by insulin tolerance test performed on both N and df/df mice after subjecting them to CR; and indicates a positive correlation of fasting glucose level with longevity [23, 35]. This physiological improvement detected in the previous and present studies brought our attention to the effects of CR in main insulin target organs such as skeletal muscle and epididymal adipose tissue[36, 37].

Both of these are major insulin responsive organs, however we observed a greater effect of CR on the expression of genes involved in the insulin signaling pathway in epididymal adipose tissue than in skeletal muscle. Whole body insulin sensitivity depends on the regulatory mechanism of insulin signaling pathway genes in diverse insulin target tissues and the interaction between these tissues in response to different diets or mutations that alter insulin signaling [38, 39].

Expression of GHR mRNA was increased in df/df-CR animals compared to df/df-AL in skeletal muscle. GH is known to stimulate protein synthesis and increase efficiency of fuel utilization[40, 41]. This upregulation of GHR may, thus, represent a response to the physiological stress caused by reduced calorie intake in df/df mice and to the lack of normal regulation of GH pathway in the absence of circulating GH in these mutant mice. In contrast, N mice did not show an upregulation of GHR transcript in response to CR in skeletal muscle; presumably due to normal physiological GH levels and signaling in these animals. Interestingly, CR also increased the expression level of PPARγ mRNA in skeletal muscle only in df/df mice, which are already characterized by elevated expression of this transcription factor compared with N mice. The upregulation of PPARγ only in df/df mice may be related to the lack of active GH signal in skeletal muscle and might contribute to the maintenance of glucose homeostasis in the condition of decreased calorie intake. It may also correlate with the increased expression of PI3K and Akt1 genes in this tissue in response to CR. However, the same CR regimen did not increase the expression of GHR and PPARγ in N-CR mice compared with N-AL. However, CR did affect the expression of genes in the insulin signaling pathway further downstream by leading to an increase in the expression of Akt1, Akt2 and FOXO3 at the mRNA level. These latter effects indicate stimulation of insulin signaling genes in skeletal muscle of N-CR mice. Activation of this part of the insulin signaling pathway may represent the mechanism that partially regulates whole body insulin sensitivity in response to reduced calorie intake.

Importantly, our results reveal that CR differentially regulates expression of genes involved in insulin signaling in skeletal muscle of N mice when compared to df/df mice, similar to previous findings in the liver [30, 42]).

The analysis of the expression of the same genes in epididymal adipose tissue indicated significant interaction between diet and genotype for most of the genes, showing different responses of N and df/df mice to CR. Interestingly, in this adipose tissue depot, GHR transcript levels were not affected in df/df-CR mice, as in skeletal muscle. However, the diet increased the level of GHR mRNA in N-CR mice compared to N-AL, which may suggest increased fat mobilization only in N animals during starvation. The observed enhanced mRNA expression of IGF-1 in adipose tissue of N-CR mice confirms the activation of GH signaling pathway. In Normal animals, CR also upregulated gene expression of IR, IRS1, PI3K, FOXO1, FOXO3a, Akt1, Akt2 and GLUT4 indicating activation of the main insulin signaling pathway and presumably enhancement of insulin signal in the adipose tissue. This activation may represent an important mechanism of the major suppression of fasting blood glucose levels and the enhancement of insulin sensitivity in response to CR in the N-CR group. In contrast, in df/df mice, CR led to an increase in the gene expression of FOXO1 and GLUT4 only. This may explain much smaller suppression of fasting glucose levels in these long-living mutants, already characterized by maintaining low glucose levels. Additionally, CR enhanced the expression of PPARγ and PPARδ in both N-CR and df/df-CR groups, suggesting an important role of these transcription factors in maintaining balanced fatty acid metabolism and lipid homeostasis during periods of starvation in calorically restricted animals. Calorie restriction also caused an increase in PGC1α gene expression in adipose tissue of N-CR mice. This suggests enhanced mitochondria biogenesis in N animals subjected to reduced calorie intake. The major alterations in insulin signaling pathway in N-CR but not df/df-CR mice in the present study may be explained by the differences in the size of the adipocytes. df/df-AL adipocytes were much smaller than adipocytes in N-AL animals and CR decreased the size of adipocytes in N-CR but not in df/df-CR animals. The size of adipocytes is related to their function and it is well accepted that smaller adipocytes are characterized by improved function and produce more insulin-sensitizing adiponectin [43, 44]. Ames dwarf mice are known to have higher level of circulating adiponectin than N littermates. Our study also showed that CR increased the level of adiponectin in N-CR mice, bringing it to the level maintained by df/df-AL. This again, closely corresponds to the alterations in the size of adipocytes in N-CR animals. There were no additional alterations in adiponectin levels in df/df-CR mice, reflecting a lack of change in the size of the adipocytes. It is believed that adiponectin is mostly produced and released into circulation by adipocytes, but several studies show the expression, production and role of adiponectin in skeletal muscle [45]. Our analysis of adiponectin gene expression indicated no difference in mRNA adiponectin levels between N-AL and df/df-AL mice, and a significant increase caused by CR only in N-CR animals. However, the protein levels of adiponectin were surprisingly lower in adipose tissue of df/df-AL and df/df-CR mice compared to N-AL animals and it was even more surprising that CR suppressed adiponectin levels in adipose tissue of N animals. The levels of adiponectin in epididymal fat did not correlate with the observed plasma levels suggesting that epididymal fat may not be the main source of circulating adiponectin in these animals, but rather subcutaneous adipose tissue can represent main source of circulating adiponectin in these animals. Also higher insulin sensitivity and healthier metabolism in df/df and CR animals could preserve the usage and breakdown of adiponectin in plasma. This would allow maintaining higher level of adiponectin in plasma from CR and df/df mice with concomitant suppression of production of adiponectin in adipose tissue. On the other hand, both genotype and diet effects on adiponectin levels in the skeletal muscle were very different from those observed in the epididymal fat. Although the measurements of mRNA levels indicated lower expression of adiponectin in df/df-AL mice than in N-CR animals, the protein levels showed opposite alterations. Both df/df-AL and df/df-CR mice had higher skeletal muscle expression of adiponectin than their N-AL littermates. Additionally, CR increased the level of this anti-inflammatory protein in both skeletal muscle and plasma of N-CR mice relative to N-AL animals. This unexpected observation may suggest that during CR while the adipose tissue is mobilized for the supply of energy substrates, the skeletal muscle takes over the production of adiponectin, likely in connection with the improved insulin sensitivity. This may represent important shift in responses of the organs to reduction of calorie intake leading to maintenance of healthy glucose homeostasis. However, as there is more evidence that adipoinectin is expressed and produced in skeletal muscle it is not well studied if skeletal muscle is also secreting this protein to circulation or the expression and production of this adipokine has only local physiological function.

In summary, this study showed that CR exerts differential effects on the expression of genes in the insulin signaling pathway in skeletal muscle and epididymal adipose tissue in the long-living df/df mice as compared to genetically normal animals. It also showed that some of the characteristics of df/df mice mimic those of N animals exposed to CR, while the underlying mechanisms are not identical. Additionally our data suggest existence of an important cross talk between skeletal muscle and the adipose tissue in the regulation of adiponectin production, insulin sensitivity and longevity by nutritional signals.

## MATERIAL AND METHODS

### Experimental Design and Animals

Ames dwarf mice and their normal siblings were housed with a 12-hour light and dark cycle at a temperature between 20°C and 24°C. The mice were fed ad libitum (AL) with a nutritionally balanced diet (Rodent Laboratory Chow 5001; not autoclaved; 23.4% protein, 4.5% fat, 5.8% crude fiber; LabDiet PMI Feeds, Inc., St. Louis, MO).

At the age of 3 months, df/df and N male mice were randomly divided into four experimental groups, with 10 animals per group: normal mice with unlimited food access (N-AL), normal mice subjected to 30% CR (N-CR), Ames dwarf mice with unlimited food access (df/df-AL), Ames dwarf mice subjected to 30% CR (df/df-CR).

Calorie restriction was started at the age of 3 months and CR animals, for the first week, received 90% of the daily food consumption of the AL fed control group. In the second week they were fed 80% and in the third week until the end of the study, CR mice were maintained on 70% of daily food consumption of the AL group. Calorie restricted animals were fed at approximately 5 pm every day.

The food consumption was calculated and adjusted weekly according to age matched littermate controls. After the animals reached an age of 12 months (approximately 8 months of CR continuation) the day before sacrifice CR animals received food as usual and after approximately 2 hours all left food was removed from the cages of CR and AL animals for overnight fasting. Animals were fasted for approximately 14-18 hours. Tissue collection was started at approximately 8 a.m. by analyzing fasting blood glucose using a standard glucometer and body weight data was recorded. Animals were then anesthetized with isoflurane, bled by cardiac puncture and sacrificed by cervical dislocation. Plasma, epididymal adipose tissue, and hind-limb muscles were collected and snap frozen in liquid nitrogen and stored at −80°C for further analysis. All experiments were approved by SIU Laboratory Animal Care Committee [46].

### RNA Extraction

Approximately 50 mg of frozen skeletal muscle and epididymal adipose tissue was used for RNA extraction with miRNeasy^®^ Mini Kit (Qiagen, Valencia, CA, USA) as described in the manufacturer's protocol. The tissues were homogenized in QIAzol^®^ Lysis Reagent using 15 mg Zirconium oxide beads (Ø 1.00 mm for skeletal muscle and Ø 0.50 mm for adipose tissue) with the Bullet Blender Homogenizer BBX24 (Next Advance, Averill Park, NY, USA). Samples of adipose tissue required an additional step of processing by centrifuging for 10 min. at 12,000g at 4°C after which the supernatant was carefully transferred into new tubes. RNA samples were eluted from the columns with 40 μl of RNase-free water. The concentration and purity of each sample were determined using the Gen 5 plate reader (Epoch^TM^ Mircoplate Spectrophotometer, BioTek, Winooski, VT, USA). RNA samples with an absorption spectrum of A260/A280 < 1.9 had to be extracted again. RNA samples were frozen at −80°C until needed for analysis.

cDNA Synthesis. Total RNA extracted from skeletal muscle and epididymal adipose tissue was used to synthesize complementary DNA with iScript^TM^ cDNA Synthesis Kit (BioRad, Hercules, CA, USA) according to the protocol provided. 1 ug RNA was used for each reaction.

### Quantitative Real-Time Polymerase Chain Reaction (qPCR)

Real-time polymerase chain reaction (RT-PCR), for analysis of transcript levels, was carried out using Fast SYBR^®^ Green Mastermix (Applied Biosystems, Foster City, CA, USA), specific forward and reverse primers (Table 1) on 7900HT Fast Real-Time PCR System (Applied Biosystems) with the following run conditions: enzyme activation at 95°C for 20 seconds, followed by 45 repeated temperature cycles which are segmented in three stages with denaturation at 95°C for 1 second, annealing at 62°C for 20 seconds and elongation at 62°C for 20 seconds. The relative gene expression was analyzed and calculated as previously reported [47].

**Table 1:** Sequence of Primers used for qPCR

Gene	Primers	Sequence
**B2M**	Forward Primers Reverse Primers	5′-AAG TAT ACT CAC GCC ACC CA-3′ 5′-CAG GCG TAT GTA TCA GTC TC-3′
**Adiponectin**	Forward Primers Reverse Primers	5′-CTT CTT GGT CCT AAG GGT GA-3′ 5′-CGA TAC ACA TAA GCG GCT TC-3′
**Akt1**	Forward Primers Reverse Primers	5′-CCG CCT GAT CAA GAT GAC AGC A-3′ 5′-TGA TCC ATG CGG GGC TTC TG-3′
**Akt2**	Forward Primers Reverse Primers	5′-GAG GAC CTT CCA TGT AGA CT-3′ 5′-CTC AGA TGT GGA AGA GTC AC-3′
**FOXO1**	Forward Primers Reverse Primers	5′-TAT TGA GCG CTT GGA CTG TG-3′ 5′-TGG ACT GCT CCT CAG TTC CT-3′
**FOXO3a**	Forward Primers Reverse Primers	5′-TCC CAG ATC TAC GAG TGG ATG G-3′ 5′-CCT TCA TTC TGA ACG CGC AT-3′
**GHR**	Forward Primers Reverse Primers	5′-AGG TCT CAG GTA TGG ATC TTT GTC A-3′ 5′-GCC AAG AGT AGC TGG TGT AGC CT-3′
**GLUT4**	Forward Primers Reverse Primers	5′-ATT GGC ATT CTG GTT GCC CA-3′ 5′-GGT TCC GGA TGA TGT AGA GGT A-3′
**IGF-1**	Forward Primers Reverse Primers	5′-CTG AGC TGG TGG ATG CTC TT-3′ 5′-CAC TCA TCC ACA ATG CCT GT-3′
**IR**	Forward Primers Reverse Primers	5′-GTT CTT TCC TGC GTG CAT TTC CCA-3′ 5′-ATC AGG GTG GCC AGT GTG TCT TTA-3′
**IRS-1**	Forward Primers Reverse Primers	5′-AGC CCA AAA GCC CAG GAG AAT A-3′ 5′-TTC CGA GCC AGT CTC TTC TCT A-3′
**PGC1α**	Forward Primers Reverse Primers	5′-TAC GCA GGT CGA ACG AAA CT-3′ 5′-TGC TCT TGG TGG AAG CA-3′
**PI3K**	Forward Primers Reverse Primers	5′-TAG CTG CAT TGG AGC TCC TT-3′ 5′-TAC GAA CTG TGG GAG CAG AT-3′
**PPARγ**	Forward Primers Reverse Primers	5′-GTC AGT ACT GTC GGT TTC AG-3′ 5′-CAG ATC AGC AGA CTC TGG GT-3′
**PPARδ**	Forward Primers Reverse Primers	5′-CTC GAG TAT GAG AAG TGC GA-3′ 5′-CAT CCG TCC AAA GCG GAT AG-3′

### Protein Extraction

Protein was extracted from approximately 150 mg of frozen skeletal muscle and epididymal adipose tissue. Tissue samples were homogenized as described above, in Tissue Protein Extraction Reagent (T-PER) with Inhibitor Cocktail (protease and phosphatase inhibitor) (Thermo Scientific, Waltham, MA, USA). Homogenates were centrifuged for 10 min. at 13,200 g at 4°C, after which the middle fat-free layer was carefully transferred to a new tube which was then centrifuged again. BCA assay was used for protein quantificarion as per the manufacturer's protocol. Each sample was diluted 10 times in T-Per.

### Analysis of adiponectin protein levels by Enzyme-linked Immunosorbent Assay (ELISA)

Adiponectin protein levels in skeletal muscle, epididymal adipose tissue and plasma were analyzed using mouse adiponectin ELISA kit (Invitrogen, Grand Island, NY, USA) according to the manufacturer's protocol. To investigate the adiponectin levels in adipose tissue, the proteins were first adjusted to a concentration of 0.5 μg/μL in a total volume of 60 μL in the same extraction buffer (T-PER); then they were diluted to a concentration of 0.0025 μg/μL using the (1X) diluent buffer from the provided kit. The other steps were followed as indicated in the protocol provided.

### Histology

Epididymal adipose tissue from N-AL, df/df-AL, N-CR and df/df-CR was fixed in 4% phosphate-buffered paraformaldehyde (pH 7.4), embedded in paraffin, sectioned and stained with hematoxylin and eosin.

### Statistical Analysis

Statistical analyses were performed by using two-way analysis of variance (ANOVA) followed with Student's t-test in each group to analyze the effects of the diet within genotypes and genotypes within diets. Alpha was defined as 0.05 and values of *p* < 0.05 display a statistical significance. Results are indicated as mean ± standard error of the mean (SEM). All statistics and graphs were performed by using Prism 5 (GraphPad Software, San Diego, CA, USA).
